# Challenges in the prenatal and post-natal diagnosis of mediastinal cystic hygroma: a case report

**DOI:** 10.1186/1752-1947-2-256

**Published:** 2008-08-01

**Authors:** Sarfraz Ahmed Nazir, Syed Arsalan Raza, Sheraz Nazir, William Sherwood, Colene Bowker, Kokila Lakhoo

**Affiliations:** 1 Department of Radiology, John Radcliffe Hospital, Oxford, OX3 9DU, UK; 2 Department of Cardiology, John Radcliffe Hospital, Oxford, OX3 9DU, UK; 3 Department of Paediatric Surgery, John Radcliffe Hospital, Oxford, OX3 9DU, UK; 4 Department of Pathology, John Radcliffe Hospital, Oxford, OX3 9DU, UK

## Abstract

**Introduction:**

Cystic hygroma is a benign congenital neoplasm that mostly presents as a soft-tissue mass in the posterior triangle of the neck. Pure mediastinal lesions are uncommon; the vast majority are asymptomatic and are an incidental finding in adulthood. The diagnosis is often made intra- or postoperatively. Prenatal identification is exceptional and post-natal diagnosis also proves challenging.

**Case presentation:**

We report one such case that was mistaken for other entities in both the prenatal and immediate post-natal period. Initial and follow-up antenatal ultrasound scans demonstrated a multicystic lesion in the left chest, and the mother was counselled about the possibility of her baby having a congenital diaphragmatic hernia. Initial post-natal chest radiographs were reported as normal. An echocardiogram and thoracic computed tomography scan confirmed a complex multiloculated cystic mediastinal mass. The working diagnoses were of a mediastinal teratoma or congenital cystic adenomatous malformation. At operation, the lesion was compressed by the left lung and was found to be close to the left phrenic nerve, which was carefully identified and preserved. After excision, histopathological examination of the mass confirmed the diagnosis of cystic hygroma. Postoperative dyspnoea was observed secondary to paradoxical movement of the left hemidiaphragm and probable left phrenic neuropraxia. This settled conservatively with excellent recovery.

**Conclusion:**

Despite the fact that isolated intrathoracic cystic hygroma is a rare entity, it needs to be considered in the differential diagnosis of foetal and neonatal mediastinal masses, particularly for juxtadiaphragmatic lesions. The phrenic nerve is not identifiable on prenatal ultrasound imaging, and it is therefore understandable that a mass close to the diaphragm may be mistaken for a congenital diaphragmatic hernia because of the location, morphology and potential phrenic nerve compression. Post-natal diagnosis may also be misleading as many mediastinal cystic masses have similar appearances on imaging. Therefore, as well as cystic architecture, special consideration needs to be given to the anatomical location and effect on local structures.

## Introduction

Cystic hygromas are slow-growing benign tumours resulting from a developmental anomaly of the lymphatic system. They are reported to occur in between 1 in 6000 and 1 in 16,000 live births. They can occur anywhere in the body, but 75% involve the posterior neck, 20% the axilla and 1% the mediastinum, groin and retroperitoneum [1-3]. Isolated mediastinal lesions are rare. Prenatal and post-natal recognition of mediastinal cystic hygromas can prove equally difficult. We highlight a case where imaging entertained several diagnoses until the lesion was definitively identified postoperatively. We offer an overview of the role of imaging and suggest that both the local anatomy and the organisation of the cystic structure be borne in mind in the assessment of these mediastinal masses.

## Case presentation

A 27-year-old Sri Lankan woman presented to our hospital in week 38 of her first pregnancy. An initial antenatal ultrasound scan (USS) at 37 weeks at a peripheral hospital demonstrated a multicystic lesion in the left side of the baby's chest with mediastinal shift. The stomach was found to be appropriately placed below the diaphragm, but the lesion had the appearances of a loop of small bowel. A repeat scan at 38 weeks suggested that the spleen was intermittently in the left chest. The mother was counselled while at the regional hospital about the possibility of her baby having a congenital diaphragmatic hernia (CDH).

**Figure 1:**
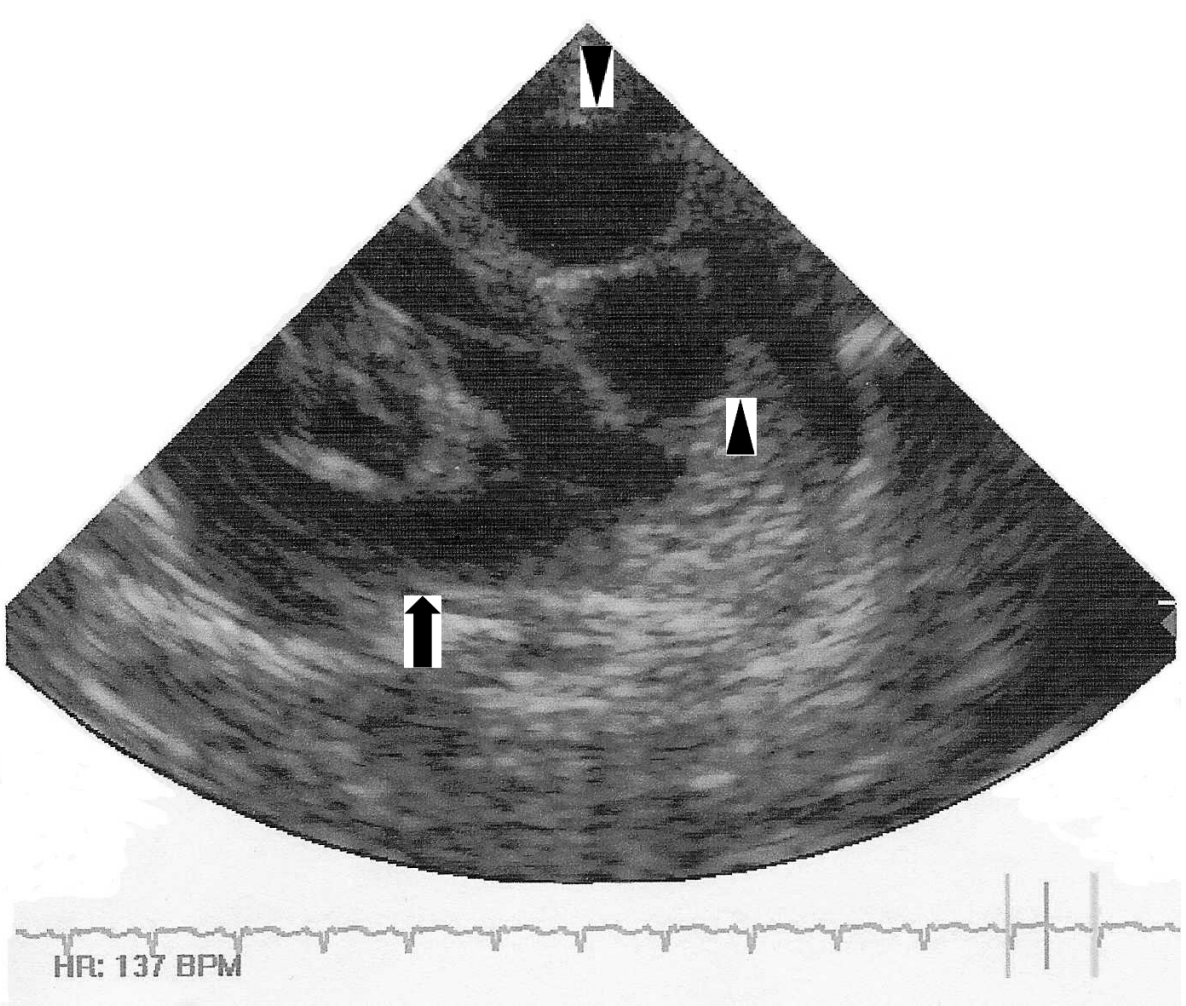
Post-natal echocardiogram showing a multiloculated, cystic mass (arrowheads) separately from the heart (arrow).

**Figure 2:**
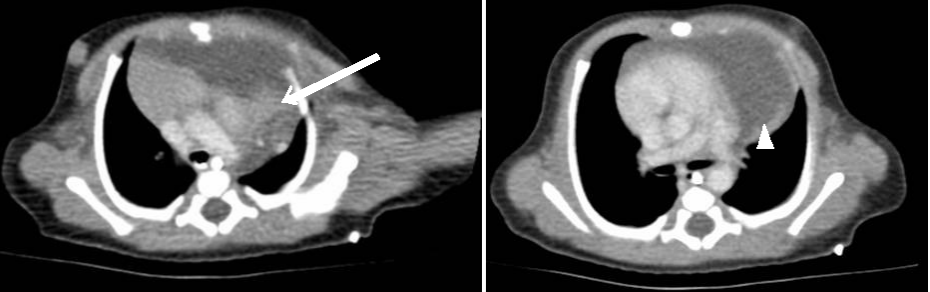
**Contrast-enhanced computed tomography scan**. Superiorly, this revealed a mixed attenuation, mainly cystic mass with a solid component within its left lateral aspect (arrow). More inferiorly, the lesion was of a more fluid density (arrowhead). These characteristics suggested a teratoma or congenital cystic adenomatoid malformation rather than cystic hygroma as the working diagnosis.

The child was born at term by forceps-assisted delivery and was immediately intubated. He required minimal ventilation, and an initial chest X-ray was reported as normal. After extubation, the child exhibited intermittent signs of respiratory distress. An echocardiogram revealed a multiloculated cystic mediastinal mass (Figure 1). A subsequent thoracic computed tomography (CT) scan showed a mainly cystic mass within the anterior mediastinum with some solid components in the left lateral aspect, which was believed to most likely represent a mediastinal teratoma or a congenital cystic adenomatous malformation (Figure 2).

**Figure 3:**
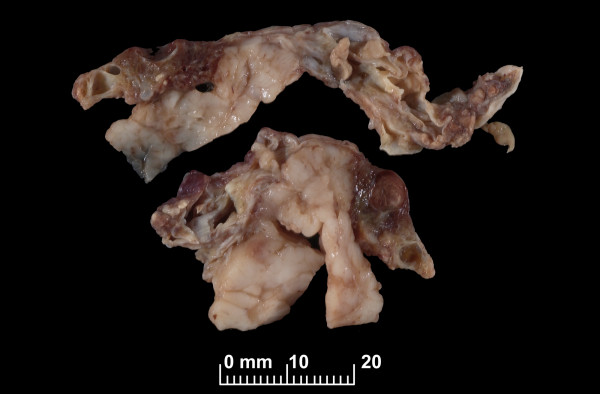
Gross specimen removed from the patient was confirmed on histopathological testing to be an intrathoracic cystic hygroma.

The lesion was subsequently completely excised via a median sternotomy approach and was indeed a multiseptated, multicystic mass with solid components. It was adherent to the thymus and right pleura in the superior mediastinum, the great vessels and the pericardium, and traversed the left pleural cavity to the left hemidiaphragm. The left lung was compressed by the lesion, which was lying very close to the left phrenic nerve. This was carefully identified and preserved. Gross and histopathological examination confirmed the diagnosis of cystic hygroma (Figure 3).

In the immediate postoperative period, dyspnoea was observed when feeding. An USS showed paradoxical movement of the left hemidiaphragm, and a left phrenic neuropraxia was suspected. This was managed conservatively, and an excellent recovery was made within 3 months. The patient is symptom free at 1 year of age.

## Discussion

Redenbacker [4] first described a cystic hygroma in 1828. It is thought that it results from an early sequestration of embryonic lymphatic channels. The term 'hygroma' describes an endothelial-lined mass consisting of small-to-medium-sized lumina containing lymphatic fluid, together with a mixture of loose collagen tissue, adipose tissue and, occasionally, vascular tissue. The cysts may be unilocular, but more often the structure contains multiple cysts infiltrating the surrounding structures and distorting the local anatomy.

Most are found in the cervical region presenting as an obvious swelling, which can be transilluminated. Mediastinal cystic hygromas are usually extensions of cervical hygromas, as 1% to 2% of cervical cystic hygromas have mediastinal extensions [5].

Isolated intrathoracic cystic hygroma is a rare finding; less than 1% of all cystic hygromas are purely mediastinal in origin. In the majority of cases, these are located in the anterior mediastinum and reveal themselves after a period of latency because of their inherent slow growth. Patients remain completely asymptomatic and so the diagnosis is not made until adulthood, usually incidentally on routine plain chest radiography [6]. Larger intrathoracic lesions tend to envelop neighbouring structures such as the trachea, oesophagus, large blood vessels and heart. In young infants with larger pure mediastinal cystic hygromas, a varying degree of respiratory compromise is the most common presenting symptom, usually secondary to extrinsic compression of the airway. There may be physical compression of the lung, as in this case, which may lead to respiratory distress and possible asphyxia. Rarer manifestations include dysphagia, superior vena caval syndrome, Horner's syndrome, phrenic nerve paresis or haemoptysis.

A prenatal diagnosis of mediastinal cystic hygromas is difficult [7]. The usual pathologies considered in the differential diagnosis of such lesions are listed in Table 1. Traditionally, ultrasonography has been used as the primary screening method for prenatal diagnosis, especially in cervical lesions. CT is avoided because of issues relating to radiation exposure to mother and foetus. Magnetic resonance imaging (MRI) may play a role in prenatal imaging, as amongst other things it provides early comprehensive information about both the anatomy and the extension of the tumour [8]. There is, therefore, a case for advocating an MRI scan for all lesions that are equivocal in prenatal screening.

**Table 1:** Differential diagnosis of cystic mediastinal chest masses

Congenital diaphragmatic hernia
Congenital cystic adenomatoid malformation
Cystic hygroma
Pulmonary sequestration
Bronchogenic and neurogenic cysts
Congenital lobar emphysema
Castleman's lymphoma
Thymic cyst or cystic thymoma
Cystic teratoma
Pericardial cyst

Our case illustrates the complexities of prenatal diagnosis. On prenatal sonography, if the stomach is visualized in its correct anatomical location in the abdomen, any mass in the left chest is unlikely to be due to a CDH. In the above case, the stomach was visualized below the diaphragm on the prenatal ultrasound examination. In what would have been an atypical presentation, the diagnosis of a CDH was still entertained because the multicystic morphology of the foetal chest mass was thought to represent a herniated intestinal loop through the diaphragm. The mass was noted to stretch from the superior mediastinum to the dome of the left hemidiaphragm. The authors appreciate the fact that the phrenic nerve is not identifiable on prenatal ultrasound screening. However, the possibility of sporadic compression of the left phrenic nerve resulting from its anatomical location of the lesion, coupled with the cystic nature of the lesion, would be reason enough for the lesion to be mistaken for bowel and hence a CDH on antenatal scanning. On a separate and more general note, colour Doppler blood flow was not used in prenatal assessment to evaluate whether the foetal chest mass had a vascular component. When faced with a mediastinal mass, we strongly advocate its use to see whether blood flow is present. It is valuable in narrowing the differential diagnosis and will help in diagnosing pulmonary sequestration or a vascular tumour such as a haemangioma.

Most cases of cystic hygromas are diagnosed post-natally, and as is borne out in the present case, even this can prove challenging as many mediastinal cystic masses have similar appearances on imaging [9]. A diagnostic clue to detection is mediastinal widening noted on routine chest radiography, but lesions are better evaluated with a CT scan [10]. CT is helpful in confirming the cystic nature of the mass and the anatomical location. It is also particularly useful in planning the potential surgical approach because of the frequent involvement of local vascular, visceral and neural structures [9,10]. The most common characteristic on CT is a well-capsulated, smoothly marginated and cystic mass, with no evidence of calcification [11]. However, as this case aptly demonstrates, thoracic CT may show a complex heterogeneous mass with varied attenuation values within the lesion [4]. MRI can be used as a radiation dose-saving modality to demonstrate the relationship of the mass with surrounding structures or as an adjunct to other imaging methods. Typically, T1-weighted magnetic resonance images reveal a mass returning a mainly low-intensity signal, but a faint high-intensity signal may represent mucoid matter within it. T2-weighted MRI shows a mostly high-intensity signal. However, a recent series reported various signal characteristics of such lesions and concluded that the diagnosis of cystic hygroma on MRI image findings can be difficult [10,12]. We elected not to perform MRI in this case because of the combination of respiratory distress and the suspicion of a neoplastic lesion. It was only after the tumour was surgically excised that the diagnosis of a mediastinal cystic hygroma was made.

Cervical cystic hygromas have been known to undergo spontaneous regression because of infection and the associated fibrotic process. Similar experience with intrathoracic lesions has not been observed. The role of non-surgical therapeutic strategies remains controversial. Aspiration of cysts is fraught with difficulty and incurs a high risk of recurrence. Sclerotherapy has been used in the management of cervical cystic hygromas, but there is no follow-up data documenting long-term success. Notwithstanding the fact that it is acutely painful, injection of sclerosants has not been recommended for the treatment of isolated mediastinal lesions. Radiotherapy is limited as an option because of increased risks of thyroid malignancy, tracheitis, oesophagitis and injury to local neurovascular tissues. Thus, the only effective treatment of mediastinal cystic hygroma remains careful surgical excision, which can be performed in one or more stages. Care should be taken that the capsule is left intact as rupture predisposes to incomplete removal and recurrence. Complete excision may prove hazardous because of adherence of parts of the tumour to neighbouring vital structures and, therefore, is not always achievable. In such cases, it is prudent to deroof the cyst and resect the maximum amount of cyst wall, rather than risk further harm. The prognosis is extremely favourable following surgery, although regular postoperative follow-up is highly recommended. Follow-up imaging is usually by way of MRI and is performed to look for rare complications such as infection, local recurrence and fistula formation.

## Conclusion

In interpreting foetal and neonatal chest masses, mediastinal cystic hygroma should be kept in mind as a potential differential diagnosis. Masses close to the diaphragm may be problematic to diagnose, particularly if they are cystic. Even though both prenatal and post-natal imaging is unable to identify the phrenic nerve, the prospect of phrenic nerve compression should be considered as a possible consequence of the location of such masses. Therefore, careful attention should be paid to both the anatomical site and the organisation of the cystic structure.

## Abbreviations

CDH: Congenital diaphragmatic hernia; CT: Computed tomography; MRI: Magnetic resonance imaging; USS: Ultrasound scan.

## Competing interests

The authors declare that they have no competing interests.

## Consent

Written informed consent was obtained from the patient's next-of-kin for publication of this case report and any accompanying images. A copy of the written consent is available for review by the Editor-in-Chief of this journal.

## Authors' contributions

SAN liaised with all authors, obtained the radiological imaging, wrote the paper, rewrote the first draft of the discussion from the second author and edited the submission after receiving the reviewers' comments. SAR wrote the first draft of the discussion. SN obtained the echocardiogram image, located the maternal notes from the referring hospital and edited and proofread the final submission. WS provided the narrative on the clinical history and surgical details. CB provided the histopathology images and reports. KL proofread the first submission and edited appropriately.
